# Cost-utility of ranibizumab versus aflibercept for treating Greek patients with visual impairment due to diabetic macular edema

**DOI:** 10.1186/s12962-016-0056-1

**Published:** 2016-04-14

**Authors:** Georgia Kourlaba, John Relakis, Ronan Mahon, Maria Kalogeropoulou, Georgia Pantelopoulou, Olga Kousidou, Nikos Maniadakis

**Affiliations:** EVROSTON LP, 5, Chatzigianni Mexi, 115 28 Athens, Greece; Department of Health Services Organization, National School of Public Health, Athens, Greece; Novartis Ireland Limited, Dublin, Ireland; Novartis Hellas SACI, Athens, Greece

**Keywords:** Ranibizumab, Aflibercept, Diabetic macular edema, Cost-effectiveness

## Abstract

**Background:**

To conduct a cost-utility analysis of ranibizumab versus aflibercept for the treatment of patients with visual impairment due to diabetic macular edema (DME) in the Greek setting.

**Methods:**

A Markov model was adapted to compare the use of ranibizumab 0.5 mg (pro re nata-PRN and treat and extend-T&E) to aflibercept 2 mg (every 8 weeks after five initial doses) in DME. Patients transitioned at a 3-month cycle among nine specified health states (including death) over a lifetime horizon. Transition probabilities, utilities, as well as DME-related mortality were extracted from relevant clinical trials, a network meta-analysis and other published studies. The analysis was conducted from payer perspective and as such only costs reimbursed by the payer were considered (year 2014). The incremental cost per quality-adjusted life year (QALY) gained and the net monetary benefit was the main outcome measures.

**Results:**

Τhe use of PRN and T&E ranibizumab regimens were shown to be cost saving comparing to aflibercept (by €2824 and €22, respectively), and more beneficial in terms of QALYs gained (+0.05) and time without visual impairment (0.031 and 0.034 years), thereby dominating aflibercept. Moreover, ranibizumab used as PRN or T&E resulted in a net monetary benefit of €3984 and €1278, respectively.

**Conclusions:**

Both PRN and T&E ranibizumab regimens were more beneficial and less costly compared to aflibercept for the management of DME. Hence, ranibizumab seems to be a dominant option for the treatment of visual impairment due to DME in the Greek setting.

**Electronic supplementary material:**

The online version of this article (doi:10.1186/s12962-016-0056-1) contains supplementary material, which is available to authorized users.

## Background

Diabetic retinopathy (DR) and diabetic macular edema (DME) are microvascular complications diagnosed in patients with diabetes mellitus [1]. Diabetic retinopathy represents a complication caused by damage to the blood vessels of the light-sensitive tissue at the back of the eye (retina) [2]. Diabetic macular edema occurs in a subset of population with DR; it is caused by thickening of tissue within the macula (the central area of the retina) as a result of fluid leaking from the blood capillaries [3]. This alteration in the structure of the macula disrupts the function of the retina and when it affects the centre of the macula, it may have a sudden and debilitating impact on visual acuity (VA), eventually leading to blindness [1].

Risk factors for the development of DME include longer duration of diabetes, progression in retinopathy, poor glucose control and hyperlipidemia [4, 5]. Epidemiological studies estimate that the overall prevalence of DME in the United States (US) and Europe ranges from 0.85 to 12.3 %, depending on type of diabetes (one or two), insulin versus non-insulin dependence, and duration of disease (years since diagnosis) [6].

Due to visual impairment (VI), and an imminent blindness, the ability of patients to manage their disease as well as the underlying diabetes is reduced [6]. Indeed, the disease is associated with a significant impact on health-related quality of life (HRQoL) in patients with diabetes [7–9]. In a US study measuring the utilities related with treatments and complications of diabetes, DR was associated with a utility of 0.53, whereas blindness had a mean utility of 0.38, ranked as the third lowest among all health states studied, following major stroke (0.31) and end-stage renal disease (0.35) [10]. However, this negative impact on patients’ perceived functional status and quality of life is considered to attenuate with treatment [9].

Consequently, there is a profound economic burden for patients and the society overall. DME is the leading cause of blindness among working-age populations in most developed countries [11–13]. Moreover, DME patients are associated with higher rates of resource use (e.g. doctor visits, hospitalizations, diagnostic modalities, treatments and maintenance medications) compared to diabetic patients without retinal diseases or with other types of diabetic retinopathy [14–16]. According to Happich et al., DME patients in Germany use almost twice the medical resources of patients with mild or moderate non-proliferative DR [17]. Similarly, Shea et al. reported that Medicare costs for DME patients in the US were more than 30 % higher than for diabetic patients without retinal disease at 1 and 3 years after diagnosis, while inpatient costs constituted almost half of the total costs [14].

For decades, standard treatments for DME have been based on intensive control of systemic metabolic factors as well as photocoagulation providing improved outcomes in large randomized clinical trials (RCTs) [18]. Notwithstanding, visual loss continues to increase in many patients despite the aforementioned therapies, giving impetus to research for new DME pharmacotherapies. Currently, both intravitreal corticosteroids and intravitreal anti-vascular endothelial growth factor (VEGF) agents are widely used in clinical settings.

VEGF-A is a major mediator of increased vascular endothelial permeability and associated retinal damage in DME [19–21]. Ranibizumab (Lucentis^®^) and aflibercept (Eylea^®^) are anti-VEGFs approved for the treatment of VI due to DME. Both ranibizumab and aflibercept are administered by intravitreal injection [22, 23]. These novel therapies may be effective treatment options for DME, but they may also impose considerable costs to the health care system and payers. Furthermore, the prolonged recession in Greece, characterized by strong health care budgetary constraints, necessitates the need to use treatments which are clinically effective but at the same time economically efficient, to maximize the value, or in other words the benefit, for the money spent in health care.

For this reason, an economic evaluation analysis was conducted to assess the cost-utility of ranibizumab versus aflibercept for the treatment of patients with VI, due to DME, in the Greek setting. At this point, it should be noted that although Bevacizumab is another widely used anti-VEGF drug for DME, it was not considered in our analysis as it has been using without being approved (off-label use).

## Methods

A cost-utility analysis (CUA) was performed to compare ranibizumab 0.5 mg pro re nata (PRN), and ranibizumab 0.5 mg treat and extend (T&E), to 2 mg aflibercept every 8 weeks after five initial monthly doses (2q8). In this context, a Markov model, developed based on the requirements of the UK National Institute for Health and Care Excellence (NICE), was adapted to the Greek health care setting to assess the health effects (i.e. DME-related outcomes) and associated costs for each treatment strategy [24]. The incremental differences between treatment arms for each measure were determined over a lifetime horizon and the comparison was captured by the incremental cost per quality-adjusted life year (QALY) gained.

The analysis was performed from a payer’s perspective, which in this case is the Greek Health Care Insurance Fund (EOPYY) covering the vast majority of the Greek population. In this context, only direct costs reimbursed by the payer were included in the model. Costs and outcomes that occurred beyond 1 year were discounted at a 3.5 % annual rate, as recommended by NICE [25], which is also the standard practice in Greece as well as other jurisdictions.

As this study is an economic evaluation analysis and does not involve human subjects no ethics approval issues arise. Input data including human material or human data were derived from other published studies performed with the approval of an appropriate ethics committee.

### Target population

The hypothetical cohort in the model reflected the general population of patients with VI due to DME. Patients were assumed to attain similar baseline characteristics to the population included in the RESTORE trial which assessed the clinical efficacy and safety of ranibizumab 0.5 mg intravitreal injection (either as monotherapy or combined with laser therapy): focal or diffuse DME due to type 1 or type 2 diabetes mellitus; mean age of 63 years; and at least one eye with a Best Corrected Visual Acuity (BCVA) score between 78 and 39 letters, using the Early Treatment of Diabetic Retinopathy Study (ETRDS)-like VA charts at a four meters-testing distance. In addition, based on the RESTORE trial, a proportion of patients were treated in both eyes, as they had bilateral disease (22 %) [26].

### Model structure

**Fig. 1:**
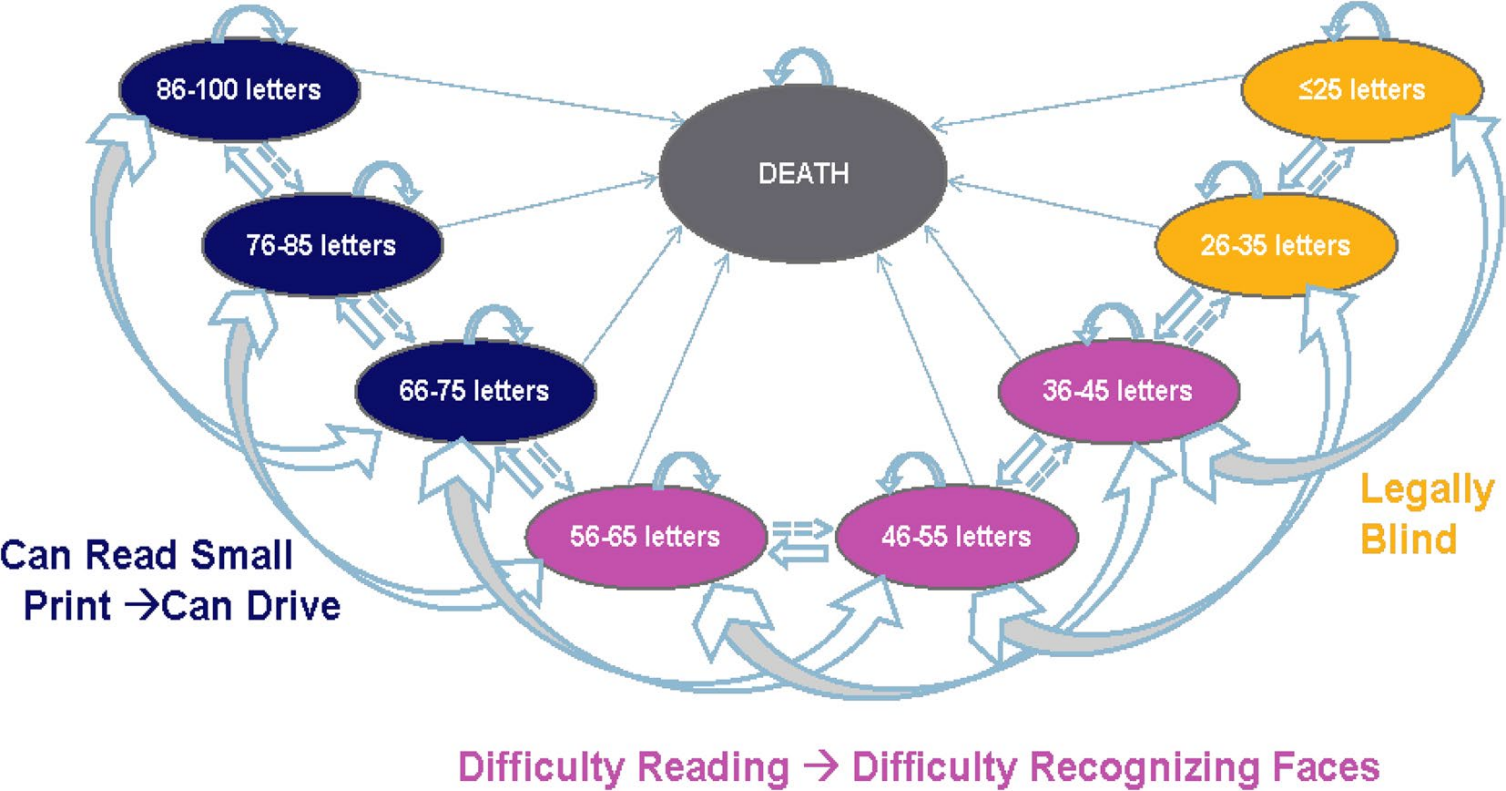
Model structure

The model structure, in its simplified form, is displayed in Fig. 1. In the model, patients transitioned during a 3-month cycle between eight BCVA-related health states—and death—over a lifetime horizon (base case). The health states were defined by BCVA groups of two lines on the ETDRS eye chart, given that a change in visual acuity of ≥10 letters (≥2 lines) is considered clinically significant [27–31]. A half-cycle correction was applied to each 3-month cycle.

The model was initiated by distributing patients in the BCVA health states as observed in the RESTORE patient population at baseline [26]. Patients were treated with the anti-VEGF treatments in the first three years, and were followed throughout their lifetime to gather all of the costs and outcomes associated with DME. Hence, the model incorporated the following variables: transition probabilities (through BCVA health states and death); BCVA-related utilities; resource utilization estimates; and unit costs for all resources and treatments. Of note, adverse events were not included in the core—and therefore the adapted—model as the incidence rates of ocular adverse events, considered both clinically and economically important, were found to be low in the RESTORE trial [32].

### Transition probabilities

For years 1–3, the actual transitions observed in the RESTORE study, specific for the level of VA, were applied to the ranibizumab PRN arm. In particular, data from the RESTORE study were used to reflect transitions in year 1 [26] and data from the RESTORE extension study were used for years 2–3 [33]. The relative efficacy (odds ratios) of ranibizumab PRN vs aflibercept—sourced from a network meta-analysis conducted by Régnier et al. [34]—was used to inform the transitions of patients treated with aflibercept in year 1. For the following years (2 and 3) the same transition probabilities as ranibizumab PRN were assumed. The relative efficacy of ranibizumab T&E vs aflibercept (used in year 1) was calculated based on the relative efficacy of ranibizumab PRN vs aflibercept [34] and the relative efficacy of ranibizumab T&E vs ranibizumab PRN [35]. As in the case of aflibercept, for years 2–3, the transition probabilities of ranibizumab PRN were used to predict the progression of patients in the ranibizumab T&E arm. Finally, from year 4 onwards (long-term progression), patients were assumed to not receive any anti-VEGF treatment, and transition probabilities were calculated using data from the Wisconsin Epidemiologic Study of Diabetic Retinopathy (WESDR) [36, 37] to capture the natural decline in BCVA without treatment, as no clinical trial evidence is available regarding effectiveness of either drug after a treatment of 2 years. The assumption of natural decline was applied equally to both drugs and as such the only effect of this assumption is to possibly underestimate the costs and effects of both drugs. A summary of inputs, regarding the transition probabilities of comparators, is provided in Table 1, while the transition probability matrices are provided in the Additional file 1.

**Table 1:** Transition probabilities used in the model

Time period	RAN PRN	RAN T&E	AFL
Year 1	RESTORE [26]	NMA [34] + RETAIN [35]	NMA [34]
Year 2	RESTORE extension [33]	Assumption (same as RAN PRN)	Assumption (same as RAN PRN)
Year 3	RESTORE extension [33]	Assumption (same as RAN PRN)	Assumption (same as RAN PRN)
Year 4+	Natural history [36, 37]	Natural history [36, 37]	Natural history [36, 37]

*NMA* network meta-analysis; *RAN PRN* ranibizumab pro re nata; *RAN T&E* ranibizumab treat and extend; *AFL* aflibercept

Due to the lack of Greek-specific data regarding mortality, in the adapted model, DME-related mortality rates were assumed to be the same as in the UK model. In the core model mortality rates resulted from multiplying the relative risk associated with developing diabetes mellitus and the relative risk associated with DME by the baseline all-cause mortality. Hence, the RR for diabetes (1.27) was sourced from Hirai et al. [38] and the RR for DME (1.93) was sourced from Mulnier et al. [39] resulting in a RR for DME related mortality of 2.45. The baseline all-cause mortality for Greece was sourced from the WHO [40].

### Utilities

The utility values used in the analysis conducted in the UK based on the NICE assessment of ranibizumab for the treatment of DME [24] were applied to each health state of the model. Since in the RESTORE trial 40.2 % of patients were treated for the better-seeing eye (BSE) and 59.6 % of patients were treated for their worse-seeing eye (WSE), utilities were specified for BSE or WSE. Moreover, in cases of patients with bilateral disease, the QALY gains from the BSE utilities were assumed. Notably, no utilities specific to DME are found in the literature. Hence, for BSE, age-adjusted utilities, originally used for wet-AMD, were sourced from the study of Czoski-Murray et al. [41].

Regarding WSE, utilities were sourced from a Canadian study conducted to assess the health state utilities associated with BCVA in patients with retinal vein occlusion (RVO) [42]. It was assumed that the RVO utilities would be relevant for the DME patient population, as these were anchored by VA. The relationship between the health utility scores of the study and the BCVA scores in the affected eye was explored using a multiple linear regression model. Subsequently, the regression model was used to estimate utilities for the 8 health states of the Markov model. The utility values in details are presented elsewhere [24].

### Resource utilization and cost inputs

Drug acquisition costs were calculated based on hospital prices, using the latest Price Bulletin available at the time of the analysis, issued by the Ministry of Health [43], and by deducting the applicable rebates to reach the final drug prices reimbursed by EOPYY. Regarding laser surgery, patients in the Greek health care system can choose either the outpatient or the hospital setting for application. For the purposes of the adapted model it was assumed that 62.5 % of patients will choose the hospital setting requiring a reimbursement of €177 as per the DRG tariffs [44]. Reimbursement for the remaining patients (37.5 %) undergoing surgery in the outpatient setting was calculated at €20.15, which corresponds to 85 % of the €23.17 cost per visit (25 % constitutes patient out-of-pocket payment) [45]. Based on the aforementioned, an average cost of €118 per laser surgery was considered in the model.

The cost of blindness was assumed to be equal to the monthly allowance of €362 that EOPYY provides to the blind in the form of social payment (€4.344 annually) [46]. Although the relevant payment is covered by the national healthcare system in Greece, it could also be considered as a “transfer payment”, and therefore excluded from an economic analysis. The administration cost of ranibizumab (and aflibercept) was set at €66 in the adapted model. This was estimated based on the specialist opinion of an expert panel, according to which 15 % of patients pay €5 as an out-patient visit to hospitals which is not reimbursed by EOPYY, 35 % of patients visit the hospital in the regimen of the one-day clinic which is reimbursed as per the relevant DRG at €80, 35 % of patients visit the hospital in the regimen of the one-day surgery which is reimbursed as per the relevant DRG at €177 and 30 % of patients visit private physicians and the physician visit is reimbursed by EOPYY at €10 [44]. Table 2 presents the cost inputs for the model specific to the Greek setting.

**Table 2:** Cost inputs used in the Greek model

Model input	Costs	Data source
Ranibizumab acquisition cost^a^	€781.52	Official price bulletin—Greek ministry of health; August 2014
Aflibercept acquisition cost	€718.83	Official price bulletin—Greek ministry of health; August 2014
Laser therapy cost (per visit)	€118	Weighted sum of the different charges as per the Greek legislature, depending on the setting of laser therapy (inpatient, outpatient)
Administration costs	€66	Weighted sum of the different charges as per the Greek legislature, depending on the setting of administration (out-patient, hospital 1 day clinic visit, hospital 1 day surgery visit, private physician visit)
Cost of blindness (annual)	€4344	Min. Dec. Π3α/Φ. 18/Γ.Π.oik.63,731; FEK 931 Β’/21-5-2008

^a^ The prices for pharmaceutical products reported in the table are the ex-factory prices that are officially published in the Price Bulletin issued by the Greek authorities. The acquisition prices incorporated in the model for the purposes of this local adaptation were the hospital prices reduced by the rebates that correspond to the third party payer, EOPYY

### Data analysis

The cost-utility of ranibizumab PRN and ranibizumab T&E compared to aflibercept was evaluated by calculating the incremental cost per QALYs gained and using a willingness-to-pay (WTP) threshold of €25,000. Health Technology Assessment is not mandatory locally and as such there is no pre-determined WTP threshold generally applied by the Greek Authorities to make decisions on the reimbursement of healthcare interventions. In this analysis, the WTP threshold was assumed to equal the equivalent willingness to pay threshold value set by NICE in euros (approx. €25 k at the time). In addition, the net monetary benefit (NMB) of ranibizumab was estimated; the NMB is equal to the incremental QALY gained multiplied by the WTP minus the incremental costs. A NMB, greater than zero, indicates that a treatment is cost-effective in health care setting.

One-way sensitivity analysis (OWSA) was performed to test the robustness of the results. The independent variables were varied within plausible pre-specified ranges in order to ascertain the key drivers of cost-effectiveness and check for the impact of uncertainty on the NMB. The following parameters have been altered: discount rate; time horizon; BSE Utilities; risk ratio of DME mortality; WSE utilities; starting age; Odds ratio Ranibizumab vs Aflibercept, rebates and costing data. The values used in the sensitivity analysis are presented in Table 3. The results are presented in the form of Tornado diagrams.

**Table 3:** Model inputs updated to the Greek healthcare setting used in sensitivity analysis

Model input	Values	Low values	High values	Variation
Time horizon	36	10	20	
Discount rate costs/outcomes	3.50 %	0 %	7.0 %	
Baseline age	63	53	73	±10 years
Utility multiplier—BSE	1	0.80	1.20	±20 %
Utility multiplier—WSE	1	0.80	1.20	±20 %
Relative risk mortality w DME	2.45	1.00	4.90	±100 %
Odds ratio ranibizumab PRN vs aflibercept month 0–3	1.5949	0.61	5.37	Standard error
Odds ratio ranibizumab T&E vs aflibercept month 0–3	1.65	0.43	6.08	Standard error
Price of ranibizumab	€656.16^a^	€492.12	€820.20	±25 % (of acquisition cost)
Price of aflibercept	€613.36^b^	€460.02	€766.70	±25 % (of acquisition cost)
Administration cost	€66.40	€49.80	€83.00	±25 %
Monitoring visit cost	€10.00	€7.50	€12.50	±25 %
BCVA ≤35 year 1	€5248.49	€3936.37	€6560.61	±25 %
BCVA ≤35 year 2+	€4866.49	€3649.87	€6083.11	±25 %

^a^ Lucentis® acquisition cost of €781.52 incorporated into the model using EOPYY rebate discount of 8%

^b^ Eylea® acquisition cost of €718.52 incorporated into the model using EOPYY rebate discount of 6.5%

*BSE* better-seeing eye; *WSE* worse-seeing eye; *DME* diabetic macular edema; *BCVA* best corrected visual acuity

The majority of input data used in the current model are subjected to variation. Therefore, in order to deal with uncertainty, a probabilistic sensitivity analysis (PSA) was performed using a second-order Monte Carlo simulation. In this analysis, a distribution was assigned around each parameter (i.e. costs, transition probabilities etc.) and the aforementioned economic and health outcomes associated with simultaneously selecting random values from those distributions were generated. Distributions were selected based on the nature of variables [47]. A cost-effectiveness acceptability curve (CEAC) was plotted, showing the proportion of simulations that are considered cost-effective at different levels of WTP per QALY gained.

## Results

### Ranibizumab PRN vs aflibercept

According to the base case results, ranibizumab PRN accumulated mean total life time costs of €12,180, whereas aflibercept accumulated costs of €15,004. In this context, savings due to the selection of ranibizumab PRN for the treatment of patients could reach €2824 over a lifetime period. Patients treated with ranibizumab PRN and aflibercept are expected to live 4.35 and 4.32 years without visual impairment (BCVA >35 letters), respectively. In addition, ranibizumab PRN generated an increment of 0.05 QALYs when compared to aflibercept (8.59 vs 8.54). Since ranibizumab PRN was able to generate savings combined with better health outcomes for the target population it is considered to dominate aflibercept in the Greek setting. The cost-effectiveness of ranibizumab PRN was further indicated by the NMB of €3984 (Table 4).

**Table 4:** Base case results—ranibizumab PRN, and ranibizumab T&E vs aflibercept

	RAN PRN	RAN T&E	AFL	RAN PRN vs AFL	RAN T&E vs AFL
Costs	€12,180	€14,982	€15,004	−€2824	−€22
Years without visual impairment (BCVA >35 letters)	4.352	4.355	4.321	+0.031	+0.034
Total QALYs	8.59	8.59	8.54	+0.05	+0.05
Cost per year without visual impairment (BCVA >35 letters)	–	–	–	−€89,807	−€653
Cost per QALY	–	–	–	Dominant	Dominant
NMB				€3984	€1278

*RAN PRN* ranibizumab pro re nata; *RAN T&E* ranibizumab treat and extend; *AFL* aflibercept; *BCVA* best corrected visual acuity; *QALY* quality-adjusted life year; *NMB* net monetary benefit

### Ranibizumab T&E vs aflibercept

Regarding the comparison of ranibizumab T&E versus aflicercept, cost savings due to the selection of ranibizumab were equal to €22 (€14,982 vs €15,004) with a QALY gain of 0.05 (8.59 vs 8.54). Moreover, an increment of 0.034 years without visual impairment (BCVA >35 letters) was attributed to ranibizumab T&E in relation to aflicercept (4.355 vs 4.321). Consequently, ranibizumab T&E is also considered a dominant option over aflicercept in the Greek setting producing a NMB of €1278. The base case results for both comparisons of the analysis are reported in Table 4 below.

### One-way sensitivity analysis

**Fig. 2:**
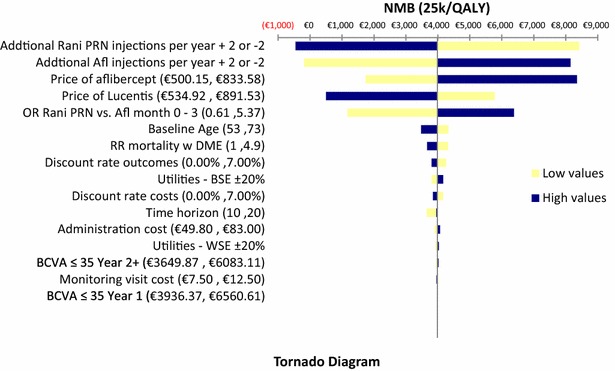
Tornado diagram ranibizumab PRN vs aflibercept. *RR* relative risk; *OR* odds ratio; *BCVA* best corrected visual acuity; *NMB* net monetary benefit; *BSE* better-seeing eye; *WSE* worse-seeing eye; *DME* diabetic macular edema

**Fig. 3:**
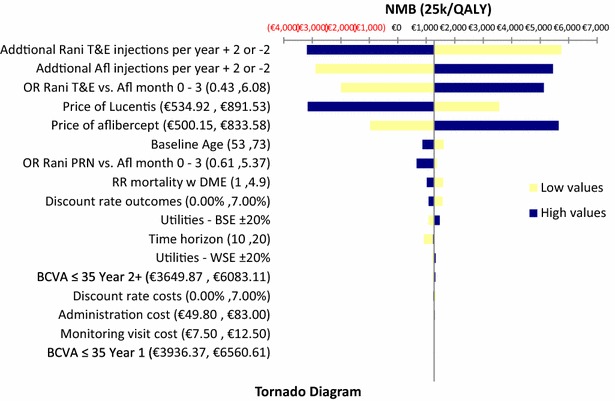
Tornado diagram ranibizumab T&E vs aflibercept. *RR* relative risk; *OR* odds ratio; *BCVA* best corrected visual acuity; *NMB* net monetary benefit; *BSE* better-seeing eye; *WSE* worse-seeing eye; *DME* diabetic macular edema

OWSA was used to explore uncertainty around input values. The price of aflibercept and ranibizumab as well as the odd ratio of ranibizumab vs aflibercept at months 0–3 were found to have the greater impact on the main results of our analysis (Figs. 2, 3). With respect to ranibizumab price, we calculated that ranibizumab ceases to be cost-effective when its reimbursed price reaches at €925.30 and €723.89 in case of PRN and T&E regimens, respectively.

### Probabilistic sensitivity analysis

**Fig. 4:**
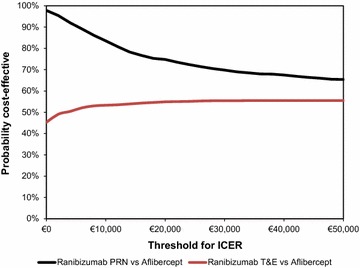
Cost effectiveness acceptability curve of ranibizumab vs aflibercept

The PSA confirms the deterministic results. The CEAC showed that at a WTP threshold of €25,000 ranibizumab PRN and T&E was almost 71 and 56 % more likely to be cost-effective over aflibercept (Fig. 4).

## Discussion

DME is a complication of DR in patients with diabetes mellitus. Moreover, if left untreated, DME causes vision loss and can eventually lead to blindness [1]. Ranibizumab and aflibercept are novel anti-VEGFs approved for the treatment of this debilitating condition [22, 23]. The analysis presented here aimed to demonstrate the most cost-effective treatment option, in terms of health outcomes and associated costs, for the Greek Health Care Insurance Fund that covers the vast majority of the Greek population.

Clinical studies have demonstrated the efficacy of ranibizumab in patients with VI due to DME over 36 months [26, 33]. A network meta-analysis by Régnier et al. [34] on the efficacy of anti-VEGF and laser photocoagulation treatments provided the relative efficacy of ranibizumab PRN vs aflibercept whereas combining data of this analysis with data from the RETAIN trial [35] allowed for the calculation of the relative efficacy of ranibizumab T&E vs aflibercept.

By adapting a UK Markov model developed for undertaking an economic evaluation that follows patients over a lifetime horizon, ranibizumab PRN and ranibizumab T&E were found to increase the years without visual impairment (BCVA >35 letters) by 0.031 and 0.034 years, respectively, when compared to aflibercept. Adjusting for preference values relative to optimal health and death for the time spent in each VA level, both ranibizumab PRN and ranibizumab T&E showed an increment of 0.05 QALYs. Moreover the ranibizumab arms managed to generate cost savings for the insurance fund in Greece. In particular, savings for ranibizumab PRN were estimated at €2824 whereas savings for ranibizumab T&E were estimated at €22. The NMB over aflibercept was estimated at €3984 for ranibizumab PRN and at €1278 for ranibizumab T&E. Hence, both options of ranibizumab are considered to dominate aflibercept in the Greek setting.

In general, the NMB of ranibizumab T&E was found to be more sensitive when altering parameters in the one-way sensitivity analysis. Nonetheless, none of the scenarios investigated questioned the cost-effectiveness of the ranibizumab arms as in all cases they dominated aflibercept. Potential changes in the NMB of ranibizumab PRN ranged from −12.75 % (starting age: 73) to +11.30 (discounting rate: 0 %) around the base case scenario. The same parameters provided the low and high range for the NMB of ranibizumab T&E. When starting age for the target population was set to 73 years the NMB was reduced to €869 (−32 %) while a discounting rate of 0 % resulted in a NMB of €1625 (+27 %).

The results of this economic analysis undertaken in the Greek setting were consistent with those published in the UK, from a health care perspective. Ranibizumab PRN and ranibizumab T&E resulted in lower lifetime costs and greater QALYs dominating aflibercept. No differences were observed in the health outcomes of the comparators (i.e. QALYs) [24].

The analysis pursued is characterized by specific drawbacks and limitations. Since the analysis was undertaken from the perspective of Greek Health Care Insurance Fund, only direct medical costs for the treatment of VI due to DME were considered. Notwithstanding, it is acknowledged that the ramifications of a DME have a wider impact generating significant indirect costs (e.g. productivity losses) that could enhance the results of our analysis. A specific drawback regarding the adaptation of this cost-effectiveness model was the fact that, due to lack of Greek-specific studies, DME-related mortality was assumed to be the same as in the UK core model. Another limitation of the study was the fact that it did not account for variation in treatment practice (i.e. the number of injections in year 1–3 was tested). Moreover, the clinical inputs of the current study were extracted from a network meta-analysis and not a head to head study. Finally, within the context of customization, it should be noted that the results refer strictly to Greece and on the basis of the present time resource and drug prices. If any of the underlying parameters change, so may the results and the conclusions of the analysis.

## Conclusions

This cost-utility analysis confirmed that, for DME patients with VI, ranibizumab 0.5 mg PRN and T&E regimens were associated with increased time without visual impairment, increased quality-adjusted survival and fewer costs versus Aflibercept under the EOPYY perspective. The model results demonstrate that ranibizumab 0.5 mg PRN and T&E consist dominant options for the treatment of VI due to DME in the Greek healthcare setting.

## Additional file

10.1186/s12962-016-0056-1 Transition probabilities.
