# Supplementary material for: Cost-utility of ranibizumab versus aflibercept for treating Greek patients with visual impairment due to diabetic macular edema
Source: Cost Eff Resour Alloc. 2016 Apr 14;14:7. doi: 10.1186/s12962-016-0056-1 (PMC4831170; doi:10.1186/s12962-016-0056-1)
Supplement: Supplementary file 1 — 10.1186/s12962-016-0056-1 Transition probabilities. [file 12962_2016_56_MOESM1_ESM.docx]

**Ranibizumab PRN Transition Probabilities:**

| Baseline to month 3 | |  |  |  |  |  |  |  |
| --- | --- | --- | --- | --- | --- | --- | --- | --- |
|  | 86-100 | 76-85 | 66-75 | 56-65 | 46-55 | 36-45 | 26-35 | 0-25 |
| 86-100 | 0.000 | 0.143 | 0.021 | 0.000 | 0.000 | 0.000 | 0.000 | 0.000 |
| 76-85 | 0.000 | 0.714 | 0.438 | 0.161 | 0.000 | 0.000 | 0.000 | 0.000 |
| 66-75 | 0.000 | 0.143 | 0.479 | 0.548 | 0.286 | 0.000 | 0.000 | 0.000 |
| 56-65 | 0.000 | 0.000 | 0.063 | 0.226 | 0.429 | 0.250 | 0.000 | 0.000 |
| 46-55 | 0.000 | 0.000 | 0.000 | 0.065 | 0.286 | 0.500 | 0.000 | 0.000 |
| 36-45 | 0.000 | 0.000 | 0.000 | 0.000 | 0.000 | 0.250 | 0.000 | 0.000 |
| 26-35 | 0.000 | 0.000 | 0.000 | 0.000 | 0.000 | 0.000 | 0.000 | 0.000 |
| 0-25 | 0.000 | 0.000 | 0.000 | 0.000 | 0.000 | 0.000 | 0.000 | 0.000 |
|  | **0.000** | **14.000** | **48.000** | **31.000** | **14.000** | **8.000** | **0.000** | **0.000** |
| month 3 to month 6 | |  |  |  |  |  |  |  |
|  | 86-100 | 76-85 | 66-75 | 56-65 | 46-55 | 36-45 | 26-35 | 0-25 |
| 86-100 | **0.333** | 0.083 | 0.000 | 0.000 | 0.000 | 0.000 | 0.000 | 0.000 |
| 76-85 | 0.667 | **0.694** | 0.304 | 0.000 | 0.000 | 0.000 | 0.000 | 0.000 |
| 66-75 | 0.000 | 0.222 | **0.587** | 0.444 | 0.000 | 0.000 | 0.000 | 0.000 |
| 56-65 | 0.000 | 0.000 | 0.109 | **0.500** | 0.300 | 0.000 | 0.000 | 0.000 |
| 46-55 | 0.000 | 0.000 | 0.000 | 0.056 | **0.500** | 0.000 | 0.000 | 0.000 |
| 36-45 | 0.000 | 0.000 | 0.000 | 0.000 | 0.200 | **1.000** | 0.000 | 0.000 |
| 26-35 | 0.000 | 0.000 | 0.000 | 0.000 | 0.000 | 0.000 | 0.000 | 0.000 |
| 0-25 | 0.000 | 0.000 | 0.000 | 0.000 | 0.000 | 0.000 | 0.000 | 0.000 |
|  | **3.000** | **36.000** | **46.000** | **18.000** | **10.000** | **2.000** | **0.000** | **0.000** |
| month 6 to month 9 | |  |  |  |  |  |  |  |
|  | 86-100 | 76-85 | 66-75 | 56-65 | 46-55 | 36-45 | 26-35 | 0-25 |
| 86-100 | 0.750 | 0.049 | 0.000 | 0.000 | 0.000 | 0.000 | 0.000 | 0.000 |
| 76-85 | 0.250 | 0.805 | 0.279 | 0.000 | 0.000 | 0.000 | 0.000 | 0.000 |
| 66-75 | 0.000 | 0.146 | 0.628 | 0.059 | 0.000 | 0.000 | 0.000 | 0.000 |
| 56-65 | 0.000 | 0.000 | 0.093 | 0.765 | 0.000 | 0.000 | 0.000 | 0.000 |
| 46-55 | 0.000 | 0.000 | 0.000 | 0.176 | 1.000 | 0.000 | 0.000 | 0.000 |
| 36-45 | 0.000 | 0.000 | 0.000 | 0.000 | 0.000 | 1.000 | 0.000 | 0.000 |
| 26-35 | 0.000 | 0.000 | 0.000 | 0.000 | 0.000 | 0.000 | 0.000 | 0.000 |
| 0-25 | 0.000 | 0.000 | 0.000 | 0.000 | 0.000 | 0.000 | 0.000 | 0.000 |
|  | **4.000** | **41.000** | **43.000** | **17.000** | **6.000** | **4.000** | **0.000** | **0.000** |
| Month 9 to month 12 | |  |  |  |  |  |  |  |
|  | 86-100 | 76-85 | 66-75 | 56-65 | 46-55 | 36-45 | 26-35 | 0-25 |
| 86-100 | 0.600 | 0.022 | 0.000 | 0.000 | 0.000 | 0.000 | 0.000 | 0.000 |
| 76-85 | 0.200 | 0.848 | 0.206 | 0.000 | 0.000 | 0.000 | 0.000 | 0.000 |
| 66-75 | 0.200 | 0.109 | 0.765 | 0.294 | 0.000 | 0.000 | 0.000 | 0.000 |
| 56-65 | 0.000 | 0.000 | 0.029 | 0.529 | 0.111 | 0.000 | 0.000 | 0.000 |
| 46-55 | 0.000 | 0.022 | 0.000 | 0.176 | 0.889 | 0.000 | 0.000 | 0.000 |
| 36-45 | 0.000 | 0.000 | 0.000 | 0.000 | 0.000 | 1.000 | 0.000 | 0.000 |
| 26-35 | 0.000 | 0.000 | 0.000 | 0.000 | 0.000 | 0.000 | 0.000 | 0.000 |
| 0-25 | 0.000 | 0.000 | 0.000 | 0.000 | 0.000 | 0.000 | 0.000 | 0.000 |
|  | **5.000** | **46.000** | **34.000** | **17.000** | **9.000** | **4.000** | **0.000** | **0.000** |

**Ranibizumab T&E Transition Probabilities:**

| Baseline to month 3 | |  |  |  |  |  |  |  |
| --- | --- | --- | --- | --- | --- | --- | --- | --- |
|  | 86-100 | 76-85 | 66-75 | 56-65 | 46-55 | 36-45 | 26-35 | 0-25 |
| 86-100 | 0.000 | 0.147 | 0.022 | 0.000 | 0.000 | 0.000 | 0.000 | 0.000 |
| 76-85 | 0.000 | 0.710 | 0.446 | 0.166 | 0.000 | 0.000 | 0.000 | 0.000 |
| 66-75 | 0.000 | 0.143 | 0.469 | 0.557 | 0.293 | 0.000 | 0.000 | 0.000 |
| 56-65 | 0.000 | 0.000 | 0.063 | 0.212 | 0.438 | 0.257 | 0.000 | 0.000 |
| 46-55 | 0.000 | 0.000 | 0.000 | 0.065 | 0.269 | 0.509 | 0.000 | 0.000 |
| 36-45 | 0.000 | 0.000 | 0.000 | 0.000 | 0.000 | 0.234 | 0.000 | 0.000 |
| 26-35 | 0.000 | 0.000 | 0.000 | 0.000 | 0.000 | 0.000 | 0.000 | 0.000 |
| 0-25 | 0.000 | 0.000 | 0.000 | 0.000 | 0.000 | 0.000 | 0.000 | 0.000 |
|  | **0.000** | **1.000** | **1.000** | **1.000** | **1.000** | **1.000** | **0.000** | **0.000** |
| month 3 to month 6 | |  |  |  |  |  |  |  |
|  | 86-100 | 76-85 | 66-75 | 56-65 | 46-55 | 36-45 | 26-35 | 0-25 |
| 86-100 | 0.333 | 0.086 | 0.000 | 0.000 | 0.000 | 0.000 | 0.000 | 0.000 |
| 76-85 | 0.667 | 0.692 | 0.312 | 0.000 | 0.000 | 0.000 | 0.000 | 0.000 |
| 66-75 | 0.000 | 0.222 | 0.579 | 0.453 | 0.000 | 0.000 | 0.000 | 0.000 |
| 56-65 | 0.000 | 0.000 | 0.109 | 0.491 | 0.308 | 0.000 | 0.000 | 0.000 |
| 46-55 | 0.000 | 0.000 | 0.000 | 0.056 | 0.492 | 0.000 | 0.000 | 0.000 |
| 36-45 | 0.000 | 0.000 | 0.000 | 0.000 | 0.200 | 1.000 | 0.000 | 0.000 |
| 26-35 | 0.000 | 0.000 | 0.000 | 0.000 | 0.000 | 0.000 | 0.000 | 0.000 |
| 0-25 | 0.000 | 0.000 | 0.000 | 0.000 | 0.000 | 0.000 | 0.000 | 0.000 |
|  | **1.000** | **1.000** | **1.000** | **1.000** | **1.000** | **1.000** | **0.000** | **0.000** |
| month 6 to month 9 | |  |  |  |  |  |  |  |
|  | 86-100 | 76-85 | 66-75 | 56-65 | 46-55 | 36-45 | 26-35 | 0-25 |
| 86-100 | 0.750 | 0.050 | 0.000 | 0.000 | 0.000 | 0.000 | 0.000 | 0.000 |
| 76-85 | 0.250 | 0.803 | 0.286 | 0.000 | 0.000 | 0.000 | 0.000 | 0.000 |
| 66-75 | 0.000 | 0.146 | 0.621 | 0.061 | 0.000 | 0.000 | 0.000 | 0.000 |
| 56-65 | 0.000 | 0.000 | 0.093 | 0.763 | 0.000 | 0.000 | 0.000 | 0.000 |
| 46-55 | 0.000 | 0.000 | 0.000 | 0.176 | 1.000 | 0.000 | 0.000 | 0.000 |
| 36-45 | 0.000 | 0.000 | 0.000 | 0.000 | 0.000 | 1.000 | 0.000 | 0.000 |
| 26-35 | 0.000 | 0.000 | 0.000 | 0.000 | 0.000 | 0.000 | 0.000 | 0.000 |
| 0-25 | 0.000 | 0.000 | 0.000 | 0.000 | 0.000 | 0.000 | 0.000 | 0.000 |
|  | **1.000** | **1.000** | **1.000** | **1.000** | **1.000** | **1.000** | **0.000** | **0.000** |
| Month 9 to month 12 | |  |  |  |  |  |  |  |
|  | 86-100 | 76-85 | 66-75 | 56-65 | 46-55 | 36-45 | 26-35 | 0-25 |
| 86-100 | 0.600 | 0.023 | 0.000 | 0.000 | 0.000 | 0.000 | 0.000 | 0.000 |
| 76-85 | 0.200 | 0.847 | 0.212 | 0.000 | 0.000 | 0.000 | 0.000 | 0.000 |
| 66-75 | 0.200 | 0.109 | 0.759 | 0.302 | 0.000 | 0.000 | 0.000 | 0.000 |
| 56-65 | 0.000 | 0.000 | 0.029 | 0.522 | 0.115 | 0.000 | 0.000 | 0.000 |
| 46-55 | 0.000 | 0.022 | 0.000 | 0.176 | 0.885 | 0.000 | 0.000 | 0.000 |
| 36-45 | 0.000 | 0.000 | 0.000 | 0.000 | 0.000 | 1.000 | 0.000 | 0.000 |
| 26-35 | 0.000 | 0.000 | 0.000 | 0.000 | 0.000 | 0.000 | 0.000 | 0.000 |
| 0-25 | 0.000 | 0.000 | 0.000 | 0.000 | 0.000 | 0.000 | 0.000 | 0.000 |
|  | **1.000** | **1.000** | **1.000** | **1.000** | **1.000** | **1.000** | **0.000** | **0.000** |

**Aflibercept Transition Probabilities:**

| Baseline to month 3 |  |  |  |  |  |  |  |  |
| --- | --- | --- | --- | --- | --- | --- | --- | --- |
|  | 86-100 | 76-85 | 66-75 | 56-65 | 46-55 | 36-45 | 26-35 | 0-25 |
| 86-100 | 0.000 | 0.095 | 0.013 | 0.000 | **0.000** | 0.000 | 0.000 | 0.000 |
| 76-85 | 0.000 | 0.763 | 0.328 | 0.108 | **0.000** | 0.000 | 0.000 | 0.000 |
| 66-75 | 0.000 | 0.143 | 0.597 | 0.432 | 0.201 | 0.000 | 0.000 | 0.000 |
| 56-65 | 0.000 | 0.000 | 0.063 | 0.396 | 0.320 | 0.173 | 0.000 | 0.000 |
| 46-55 | 0.000 | 0.000 | 0.000 | 0.065 | 0.480 | 0.385 | 0.000 | 0.000 |
| 36-45 | 0.000 | 0.000 | 0.000 | 0.000 | 0.000 | 0.442 | 0.000 | 0.000 |
| 26-35 | 0.000 | 0.000 | 0.000 | 0.000 | 0.000 | 0.000 | 0.000 | 0.000 |
| 0-25 | 0.000 | 0.000 | 0.000 | 0.000 | 0.000 | 0.000 | 0.000 | 0.000 |
|  | **0.000** | **1.000** | **1.000** | **1.000** | **1.000** | **1.000** | **0.000** | **0.000** |
| month 3 to month 6 |  |  |  |  |  |  |  |  |
|  | 86-100 | 76-85 | 66-75 | 56-65 | 46-55 | 36-45 | 26-35 | 0-25 |
| 86-100 | 0.333 | 0.054 | 0.000 | 0.000 | 0.000 | 0.000 | 0.000 | 0.000 |
| 76-85 | 0.667 | 0.724 | 0.215 | 0.000 | 0.000 | 0.000 | 0.000 | 0.000 |
| 66-75 | 0.000 | 0.222 | 0.676 | 0.334 | 0.000 | 0.000 | 0.000 | 0.000 |
| 56-65 | 0.000 | 0.000 | 0.109 | 0.610 | 0.212 | 0.000 | 0.000 | 0.000 |
| 46-55 | 0.000 | 0.000 | 0.000 | 0.056 | 0.588 | 0.000 | 0.000 | 0.000 |
| 36-45 | 0.000 | 0.000 | 0.000 | 0.000 | 0.200 | 1.000 | 0.000 | 0.000 |
| 26-35 | 0.000 | 0.000 | 0.000 | 0.000 | 0.000 | 0.000 | 0.000 | 0.000 |
| 0-25 | 0.000 | 0.000 | 0.000 | 0.000 | 0.000 | 0.000 | 0.000 | 0.000 |
|  | **1.000** | **1.000** | **1.000** | **1.000** | **1.000** | **1.000** | **0.000** | **0.000** |
| month 6 to month 9 |  |  |  |  |  |  |  |  |
|  | 86-100 | 76-85 | 66-75 | 56-65 | 46-55 | 36-45 | 26-35 | 0-25 |
| 86-100 | 0.750 | 0.031 | 0.000 | 0.000 | 0.000 | 0.000 | 0.000 | 0.000 |
| 76-85 | 0.250 | 0.823 | 0.195 | 0.000 | 0.000 | 0.000 | 0.000 | 0.000 |
| 66-75 | 0.000 | 0.146 | 0.712 | 0.038 | 0.000 | 0.000 | 0.000 | 0.000 |
| 56-65 | 0.000 | 0.000 | 0.093 | 0.786 | 0.000 | 0.000 | 0.000 | 0.000 |
| 46-55 | 0.000 | 0.000 | 0.000 | 0.176 | 1.000 | 0.000 | 0.000 | 0.000 |
| 36-45 | 0.000 | 0.000 | 0.000 | 0.000 | 0.000 | 1.000 | 0.000 | 0.000 |
| 26-35 | 0.000 | 0.000 | 0.000 | 0.000 | 0.000 | 0.000 | 0.000 | 0.000 |
| 0-25 | 0.000 | 0.000 | 0.000 | 0.000 | 0.000 | 0.000 | 0.000 | 0.000 |
|  | **1.000** | **1.000** | **1.000** | **1.000** | **1.000** | **1.000** | **0.000** | **0.000** |
| Month 9 to month 12 |  |  |  |  |  |  |  |  |
|  | 86-100 | 76-85 | 66-75 | 56-65 | 46-55 | 36-45 | 26-35 | 0-25 |
| 86-100 | 0.600 | 0.014 | 0.000 | 0.000 | 0.000 | 0.000 | 0.000 | 0.000 |
| 76-85 | 0.200 | 0.856 | 0.140 | 0.000 | 0.000 | 0.000 | 0.000 | 0.000 |
| 66-75 | 0.200 | 0.109 | 0.831 | 0.207 | 0.000 | 0.000 | 0.000 | 0.000 |
| 56-65 | 0.000 | 0.000 | 0.029 | 0.616 | 0.073 | 0.000 | 0.000 | 0.000 |
| 46-55 | 0.000 | **0.022** | 0.000 | 0.176 | 0.927 | 0.000 | 0.000 | 0.000 |
| 36-45 | 0.000 | 0.000 | 0.000 | 0.000 | 0.000 | 1.000 | 0.000 | 0.000 |
| 26-35 | 0.000 | 0.000 | 0.000 | 0.000 | 0.000 | 0.000 | 0.000 | 0.000 |
| 0-25 | 0.000 | 0.000 | 0.000 | 0.000 | 0.000 | 0.000 | 0.000 | 0.000 |
|  | **1.000** | **1.000** | **1.000** | **1.000** | **1.000** | **1.000** | **0.000** | **0.000** |

**Year 2 & 3 for all:**

| Month 12 - 15 | |  |  |  |  |  |  |  |
| --- | --- | --- | --- | --- | --- | --- | --- | --- |
|  | 86-100 | 76-85 | 66-75 | 56-65 | 46-55 | 36-45 | 26-35 | 0-25 |
| 86-100 | 0.500 | 0.026 | 0.036 | 0.000 | 0.000 | 0.000 | 0.000 | 0.000 |
| 76-85 | 0.500 | 0.684 | 0.214 | 0.000 | 0.000 | 0.000 | 0.000 | 0.000 |
| 66-75 | 0.000 | 0.289 | 0.643 | 0.333 | 0.000 | 0.000 | 0.000 | 0.000 |
| 56-65 | 0.000 | 0.000 | 0.107 | 0.444 | 0.333 | 0.000 | 0.000 | 0.000 |
| 46-55 | 0.000 | 0.000 | 0.000 | 0.222 | 0.667 | 0.000 | 0.000 | 0.000 |
| 36-45 | 0.000 | 0.000 | 0.000 | 0.000 | 0.000 | 1.000 | 0.000 | 0.000 |
| 26-35 | 0.000 | 0.000 | 0.000 | 0.000 | 0.000 | 0.000 | 1.000 | 0.000 |
| 0-25 | 0.000 | 0.000 | 0.000 | 0.000 | 0.000 | 0.000 | 0.000 | 1.000 |
|  |  |  |  |  |  |  |  |  |
| Month 15 - 18 |  |  |  |  |  |  |  |  |
|  | 86-100 | 76-85 | 66-75 | 56-65 | 46-55 | 36-45 | 26-35 | 0-25 |
| 86-100 | 1.000 | 0.029 | 0.031 | 0.000 | 0.000 | 0.000 | 0.000 | 0.000 |
| 76-85 | 0.000 | 0.882 | 0.250 | 0.125 | 0.000 | 0.000 | 0.000 | 0.000 |
| 66-75 | 0.000 | 0.088 | 0.563 | 0.250 | 0.000 | 0.000 | 0.000 | 0.000 |
| 56-65 | 0.000 | 0.000 | 0.125 | 0.500 | 0.250 | 0.000 | 0.000 | 0.000 |
| 46-55 | 0.000 | 0.000 | 0.031 | 0.125 | 0.500 | 0.000 | 0.000 | 0.000 |
| 36-45 | 0.000 | 0.000 | 0.000 | 0.000 | 0.250 | 1.000 | 0.000 | 0.000 |
| 26-35 | 0.000 | 0.000 | 0.000 | 0.000 | 0.000 | 0.000 | 1.000 | 0.000 |
| 0-25 | 0.000 | 0.000 | 0.000 | 0.000 | 0.000 | 0.000 | 0.000 | 1.000 |
|  |  |  |  |  |  |  |  |  |
| Month 18 - 21 |  |  |  |  |  |  |  |  |
|  | 86-100 | 76-85 | 66-75 | 56-65 | 46-55 | 36-45 | 26-35 | 0-25 |
| 86-100 | 0.333 | 0.077 | 0.000 | 0.000 | 0.000 | 0.000 | 0.000 | 0.000 |
| 76-85 | 0.667 | 0.795 | 0.174 | 0.111 | 0.000 | 0.000 | 0.000 | 0.000 |
| 66-75 | 0.000 | 0.103 | 0.696 | 0.444 | 0.250 | 0.000 | 0.000 | 0.000 |
| 56-65 | 0.000 | 0.026 | 0.130 | 0.333 | 0.250 | 0.000 | 0.000 | 0.000 |
| 46-55 | 0.000 | 0.000 | 0.000 | 0.111 | 0.500 | 0.500 | 0.000 | 0.000 |
| 36-45 | 0.000 | 0.000 | 0.000 | 0.000 | 0.000 | 0.500 | 0.000 | 0.000 |
| 26-35 | 0.000 | 0.000 | 0.000 | 0.000 | 0.000 | 0.000 | 1.000 | 0.000 |
| 0-25 | 0.000 | 0.000 | 0.000 | 0.000 | 0.000 | 0.000 | 0.000 | 1.000 |
|  |  |  |  |  |  |  |  |  |
| Month 21 - 24 |  |  |  |  |  |  |  |  |
|  | 86-100 | 76-85 | 66-75 | 56-65 | 46-55 | 36-45 | 26-35 | 0-25 |
| 86-100 | 0.400 | 0.075 | 0.000 | 0.000 | 0.000 | 0.000 | 0.000 | 0.000 |
| 76-85 | 0.600 | 0.725 | 0.320 | 0.000 | 0.000 | 0.000 | 0.000 | 0.000 |
| 66-75 | 0.000 | 0.200 | 0.600 | 0.500 | 0.000 | 0.000 | 0.000 | 0.000 |
| 56-65 | 0.000 | 0.000 | 0.080 | 0.375 | 0.250 | 0.000 | 0.000 | 0.000 |
| 46-55 | 0.000 | 0.000 | 0.000 | 0.125 | 0.750 | 0.000 | 0.000 | 0.000 |
| 36-45 | 0.000 | 0.000 | 0.000 | 0.000 | 0.000 | 1.000 | 0.000 | 0.000 |
| 26-35 | 0.000 | 0.000 | 0.000 | 0.000 | 0.000 | 0.000 | 1.000 | 0.000 |
| 0-25 | 0.000 | 0.000 | 0.000 | 0.000 | 0.000 | 0.000 | 0.000 | 1.000 |
|  |  |  |  |  |  |  |  |  |
| Month 24 - 27 |  |  |  |  |  |  |  |  |
|  | 86-100 | 76-85 | 66-75 | 56-65 | 46-55 | 36-45 | 26-35 | 0-25 |
| 86-100 | 0.800 | 0.100 | 0.000 | 0.000 | 0.000 | 0.000 | 0.000 | 0.000 |
| 76-85 | 0.000 | 0.800 | 0.185 | 0.000 | 0.250 | 0.000 | 0.000 | 0.000 |
| 66-75 | 0.200 | 0.075 | 0.630 | 0.000 | 0.000 | 0.000 | 0.000 | 0.000 |
| 56-65 | 0.000 | 0.025 | 0.185 | 0.667 | 0.250 | 0.000 | 0.000 | 0.000 |
| 46-55 | 0.000 | 0.000 | 0.000 | 0.333 | 0.500 | 0.000 | 0.000 | 0.000 |
| 36-45 | 0.000 | 0.000 | 0.000 | 0.000 | 0.000 | 1.000 | 0.000 | 0.000 |
| 26-35 | 0.000 | 0.000 | 0.000 | 0.000 | 0.000 | 0.000 | 1.000 | 0.000 |
| 0-25 | 0.000 | 0.000 | 0.000 | 0.000 | 0.000 | 0.000 | 0.000 | 1.000 |
|  |  |  |  |  |  |  |  |  |
| Month 27 - 30 |  |  |  |  |  |  |  |  |
|  | 86-100 | 76-85 | 66-75 | 56-65 | 46-55 | 36-45 | 26-35 | 0-25 |
| 86-100 | 0.625 | 0.053 | 0.000 | 0.000 | 0.000 | 0.000 | 0.000 | 0.000 |
| 76-85 | 0.375 | 0.842 | 0.286 | 0.091 | 0.000 | 0.000 | 0.000 | 0.000 |
| 66-75 | 0.000 | 0.105 | 0.571 | 0.364 | 0.000 | 0.000 | 0.000 | 0.000 |
| 56-65 | 0.000 | 0.000 | 0.143 | 0.455 | 0.000 | 0.000 | 0.000 | 0.000 |
| 46-55 | 0.000 | 0.000 | 0.000 | 0.091 | 0.500 | 0.000 | 0.000 | 0.000 |
| 36-45 | 0.000 | 0.000 | 0.000 | 0.000 | 0.500 | 1.000 | 0.000 | 0.000 |
| 26-35 | 0.000 | 0.000 | 0.000 | 0.000 | 0.000 | 0.000 | 1.000 | 0.000 |
| 0-25 | 0.000 | 0.000 | 0.000 | 0.000 | 0.000 | 0.000 | 0.000 | 1.000 |
|  |  |  |  |  |  |  |  |  |
| Month 30 - 33 |  |  |  |  |  |  |  |  |
|  | 86-100 | 76-85 | 66-75 | 56-65 | 46-55 | 36-45 | 26-35 | 0-25 |
| 86-100 | 0.571 | 0.048 | 0.000 | 0.000 | 0.000 | 0.000 | 0.000 | 0.000 |
| 76-85 | 0.429 | 0.810 | 0.150 | 0.000 | 0.000 | 0.000 | 0.000 | 0.000 |
| 66-75 | 0.000 | 0.143 | 0.850 | 0.375 | 0.000 | 0.000 | 0.000 | 0.000 |
| 56-65 | 0.000 | 0.000 | 0.000 | 0.500 | 0.000 | 0.000 | 0.000 | 0.000 |
| 46-55 | 0.000 | 0.000 | 0.000 | 0.125 | 1.000 | 0.333 | 0.000 | 0.000 |
| 36-45 | 0.000 | 0.000 | 0.000 | 0.000 | 0.000 | 0.667 | 0.000 | 0.000 |
| 26-35 | 0.000 | 0.000 | 0.000 | 0.000 | 0.000 | 0.000 | 1.000 | 0.000 |
| 0-25 | 0.000 | 0.000 | 0.000 | 0.000 | 0.000 | 0.000 | 0.000 | 1.000 |
|  |  |  |  |  |  |  |  |  |
| Month 33 - 36 |  |  |  |  |  |  |  |  |
|  | 86-100 | 76-85 | 66-75 | 56-65 | 46-55 | 36-45 | 26-35 | 0-25 |
| 86-100 | 0.500 | 0.050 | 0.000 | 0.000 | 0.000 | 0.000 | 0.000 | 0.000 |
| 76-85 | 0.500 | 0.775 | 0.385 | 0.000 | 0.000 | 0.000 | 0.000 | 0.000 |
| 66-75 | 0.000 | 0.125 | 0.577 | 0.000 | 0.000 | 0.000 | 0.000 | 0.000 |
| 56-65 | 0.000 | 0.025 | 0.038 | 1.000 | 0.600 | 0.000 | 0.000 | 0.000 |
| 46-55 | 0.000 | 0.025 | 0.000 | 0.000 | 0.400 | 0.000 | 0.000 | 0.000 |
| 36-45 | 0.000 | 0.000 | 0.000 | 0.000 | 0.000 | 0.500 | 0.000 | 0.000 |
| 26-35 | 0.000 | 0.000 | 0.000 | 0.000 | 0.000 | 0.000 | 1.000 | 0.000 |
| 0-25 | 0.000 | 0.000 | 0.000 | 0.000 | 0.000 | 0.500 | 0.000 | 1.000 |
|  |  |  |  |  |  |  |  |  |

**Year 4 onwards for all**

| State description | 86-100 | 76-85 | 66-75 | 56-65 | 46-55 | 36-45 | 26-35 | 0-25 |
| --- | --- | --- | --- | --- | --- | --- | --- | --- |
| 86-100 | 0.955 | 0.035 | 0.000 | 0.000 | 0.000 | 0.000 | 0.000 | 0.000 |
| 76-85 | 0.045 | 0.920 | 0.035 | 0.000 | 0.000 | 0.000 | 0.000 | 0.000 |
| 66-75 | 0.000 | 0.045 | 0.920 | 0.035 | 0.000 | 0.000 | 0.000 | 0.000 |
| 56-65 | 0.000 | 0.000 | 0.045 | 0.920 | 0.035 | 0.000 | 0.000 | 0.000 |
| 46-55 | 0.000 | 0.000 | 0.000 | 0.045 | 0.920 | 0.035 | 0.000 | 0.000 |
| 36-45 | 0.000 | 0.000 | 0.000 | 0.000 | 0.045 | 0.920 | 0.035 | 0.000 |
| 26-35 | 0.000 | 0.000 | 0.000 | 0.000 | 0.000 | 0.045 | 0.920 | 0.035 |
| 0-25 | 0.000 | 0.000 | 0.000 | 0.000 | 0.000 | 0.000 | 0.045 | 0.965 |
